# Microglial Cells: The Main HIV-1 Reservoir in the Brain

**DOI:** 10.3389/fcimb.2019.00362

**Published:** 2019-10-24

**Authors:** Clementine Wallet, Marco De Rovere, Jeanne Van Assche, Fadoua Daouad, Stéphane De Wit, Virginie Gautier, Patrick W. G. Mallon, Alessandro Marcello, Carine Van Lint, Olivier Rohr, Christian Schwartz

**Affiliations:** ^1^Université de Strasbourg, EA7292, FMTS, IUT Louis Pasteur, Schiltigheim, France; ^2^Division of Infectious Diseases, Saint-Pierre University Hospital, Université Libre de Bruxelles (ULB), Brussels, Belgium; ^3^UCD Centre for Experimental Pathogen Host Research (CEPHR), School of Medicine, University College Dublin, Dublin, Ireland; ^4^Laboratory of Molecular Virology, International Centre for Genetic Engineering and Biotechnology (ICGEB), Trieste, Italy; ^5^Service of Molecular Virology, Department of Molecular Biology (DBM), Université Libre de Bruxelles (ULB), Gosselies, Belgium

**Keywords:** HIV-1, Ctip2, microglial cells, reservoirs, latency, brain

## Abstract

Despite efficient combination of the antiretroviral therapy (cART), which significantly decreased mortality and morbidity of HIV-1 infection, a definitive HIV cure has not been achieved. Hidden HIV-1 in cellular and anatomic reservoirs is the major hurdle toward a functional cure. Microglial cells, the Central Nervous system (CNS) resident macrophages, are one of the major cellular reservoirs of latent HIV-1. These cells are believed to be involved in the emergence of drugs resistance and reseeding peripheral tissues. Moreover, these long-life reservoirs are also involved in the development of HIV-1-associated neurocognitive diseases (HAND). Clearing these infected cells from the brain is therefore crucial to achieve a cure. However, many characteristics of microglial cells and the CNS hinder the eradication of these brain reservoirs. Better understandings of the specific molecular mechanisms of HIV-1 latency in microglial cells should help to design new molecules and new strategies preventing HAND and achieving HIV cure. Moreover, new strategies are needed to circumvent the limitations associated to anatomical sanctuaries with barriers such as the blood brain barrier (BBB) that reduce the access of drugs.

## Introduction

Since the introduction of the combination antiretroviral therapy (cART) in 1996 the fatal evolution of the HIV-1 infection has stopped and became a chronic disease. However, despite continual efforts definitive cure has not been achieved. This is mainly due to the lack of efficient vaccination and to the existence of viral reservoirs. Indeed, as soon as the treatment is interrupted, a viral rebound is observed within a couple of weeks and viremia is comparable to the one encountered before the introduction of cART (Davey et al., 1999; García et al., 1999; Mata et al., 2005). Moreover, with ultrasensitive methods, we can still notice a residual viremia in patients under cART (Dornadula et al., 1999; Bouchat et al., 2016). The main reservoirs are the resting CD4+ T cells but we have now enough evidence which argues for the existence of many other cell reservoirs such as hematopoietic stem cells, dendritic cells, microglial cells and cells from the monocyte-macrophage lineage (reviewed in Marcello, 2006; García et al., 2018; Sung and Margolis, 2018). In addition, some of these reservoirs are found in sanctuaries such as the genital tract, the adipose tissue, the bone marrow and the brain. Targeting the infected cells in these reservoirs is even more difficult (Sadowski and Hashemi, 2019). The brain for instance constitutes a typical anatomical site, which has poor drug penetration and reduced immune surveillance due to the blood brain barrier (BBB). As a consequence, the genetical information exchange of the virus confined in the brain and other compartments such as the blood is poor (Canestri et al., 2010; Dahl et al., 2014). The measureable pool of latently infected, resting CD4+ T cells does not account fully for the early rebounding plasma HIV-1. It has been shown that the rebounding HIV-1 was genetically different suggesting that these viruses came from other cell reservoirs (Chun et al., 2000). In addition, the result of recent phylogenetic studies is in favor of other physiological observations suggesting that HIV-1 production in the brain is associated with the emergence of virus resistance (Smit et al., 2004; Strain et al., 2005) and with HIV-1 associated neurocognitive disease (HAND) (Salemi et al., 2005; Lamers et al., 2010). Up to 50% of patients under cART with undetectable virus in the blood suffer of less severe forms of HAND (Eggers et al., 2017). The pathogenesis of HAND is not fully understood but appears to be multifactorial: drug neurotoxicity of the cART, HIV replication in the central nervous system (CNS) from quiescent/latent infected cells, CNS inflammation and co-infection with other viruses such as hepatitis C virus (reviewed in Sutherland and Brew, 2018). Indeed production of newly synthesized viral protein such as Tat or gp120 from infected cells might lead to direct neural injury (reviewed in Rao et al., 2014). Recent reports described new mechanisms for HIV-1 Tat-mediated microglial inflammation which involves a novel miRNA-34a- NOD-like receptor C5 (NLRC5)-NFκB signaling axis or activation of the methyl CpG binding protein 2 (MEPC2)-STAT3 axis (Periyasamy et al., 2018, 2019). Activation by Tat of the microglial nucleotide-binding domain leucin-rich repeat and pyrin-containing receptor 3 (NLRP3) inflammasome also lead to the occurrence of neuroinflammation (Chivero et al., 2017). Indirect impact on the integrity of neurons is also observed during sustained chronic inflammation induced by secreting perivascular macrophages or microglial cells (reviewed in Eggers et al., 2017). Following activation these immune cells release neurotoxic host factors such as pro-inflammatory cytokines (TNFα, IFNα, IL6, IL8, IL1β) (Nolting et al., 2012; Zhou et al., 2016; Abassi et al., 2017) and chemokines (CCL2 and CCL5) (Woods et al., 2006; Zhou et al., 2016). All together these indirect evidences strongly support the existence of cellular reservoirs in the CNS. At least, three different types of infected cells have been described in the CNS referred as a compartment (Bednar et al., 2015): astrocytes, cells from the monocyte lineage (Wong et al., 2019) and microglial cells (Joseph et al., 2015). These putative true reservoirs are established very early during HIV-1 infection (within 3 to 5 days following infection) (Koenig et al., 1986; Whitney et al., 2014). It is well-documented that infection of the CNS results from transmigration of infected CD4+ cells (reviewed in Williams et al., 2012) and of infected monocytes across the BBB (Williams et al., 2012). However, these infected cells entering the CNS do not constitute an HIV reservoir since they have a very short half-life. Instead they will be the source of the infection of three long lived cell types: astrocytes, perivascular macrophages and microglial cells. Whether or not these cells constitute true cell reservoirs is still debated (Gray et al., 2014; Al-Harti et al., 2018; Vanhamel et al., 2019). Criteria for a cell reservoir are the followings: (i) presence of HIV-1 DNA integrated in the host genome of long lived cells, (ii) existence of mechanisms which allow the virus to persist for long period in latent cells which include mechanisms allowing establishment and maintenance of a latent infection and (iii) formation of replication-competent particles following activation of the reservoirs (Eisele and Siliciano, 2012). Two criteria of a true latency reservoir have been described in these cells: the presence of HIV-1 integrated DNA in long lived cells and the existence of mechanisms which allow the virus to persist for long period in latent cells (Blankson et al., 2002). On the other hand, it cannot be explored whether these cells can produce replication-competent viruses in human due to ethical and technical problems.

The astrocyte is the most abundant infected cell type in the CNS with up to 10–20% having HIV-1 DNA in the cell genome (Churchill et al., 2009). However a recent study using sensitive methods detected HIV-1 in brain macrophages and microglial cells but not in astrocytes (Ko et al., 2019). Moreover, the infection appears to be non-productive. Thus they might not constitute a true viral reservoir for HIV-1 (Gorry et al., 2003). On the contrary there is evidence that both macrophages and microglial cells are susceptible to HIV-1 infection and support productive infection (reviewed in Joseph et al., 2015). As already mentioned, the productive infection in these later cells in the CNS has been associated with HAND in humans and in animal models such as the macaques. Therefore, both macrophages and microglial cells can be considered as true reservoirs in the brain. However, their relative importance is not the same due to their differences in size and constitution in the brain. The macrophages are bone marrow-derived with a half-life of months and they do not undergo cell division (Koppensteiner et al., 2012). They are repopulated from monocytes crossing the BBB. Under a very efficient cART or by using therapeutic strategies preventing the specific subset of infected monocytes to cross the BBB we might expect in the future to drastically decrease this reservoir in the brain. The microglial cells originate from erythromyeloid progenitors in the yolk sac during embryogenesis and colonize the forming CNS (Kierdorf et al., 2013). They constitute therefore the main resident cells of the brain which can serve as brain macrophages. Due to their long half-life of years they constitute a very stable population (Réu et al., 2017). In addition unlike macrophages they can undergo cell division enabling HIV-1 to persist in the brain (Lawson et al., 1992). Moreover, a recent report suggests that microglial cells are highly susceptible to HIV-1 infection (Cenker et al., 2017). Altogether, the microglial cells might constitute one of the main HIV-1 reservoir in the brain.

In the review we will first present microglial cells and discuss why they might be the main HIV-1 cell reservoirs in the cART era. We suggest that microglial cells should be the main target in the brain in order to achieve cure. As a prerequisite it is needed to decipher the molecular mechanisms of the establishment and the maintenance of HIV-1 latency in these cells. In further section, we will discuss the therapeutic implications of these mechanisms followed by the discussion of the challenges facing when targeting microglial cells and how to circumvent these limitations.

## Microglial Cells, a Key Player During the Course of HIV-1 Infection

### The Biology of Microglial Cells

Microglial cells constitute the CNS resident innate immune cells (Bilimoria and Stevens, 2015). They are the most abundant mono nuclear macrophages which are found in the totality of the brain parenchyma. These cells appear very early during embryogenesis and derive from early myeloid precursors in the embryogenic yolk sac (Gomez Perdiguero et al., 2015; Sheng et al., 2015). Microglial cells colonize the entire brain parenchyma before the formation of the BBB and subsist by slow cell division during life (Gomez Perdiguero et al., 2015). The median rate of human microglial cell renewal is around 30% per year. In a recent report, it was estimated that microglial cells are on average 4.2 years old and most of the cells in the population regenerates throughout life (Réu et al., 2017). Contrary to the other potentially infected cells in the brain this slow cell division allows the persistence of HIV-1 in the brain throughout the life of the patient. Both resting and activated microglial cells are ramified cells with multiple branches and processes which contact neurons, astrocytes and blood vessels (reviewed in Sominsky et al., 2018). Resting cells are transformed to activated macrophage like state when sensing a change in their microenvironment (reviewed in Colonna and Butovsky, 2017). They are also very mobile and rapidly reach the site of brain damage. When activated, these cells release many cytokines, chemokines and other neurotoxic proteins. These secretory factors might contribute to the pathological neuroinflammation observed in HAND (Hong and Banks, 2015). Many other diseases have been associated with pathological neuroinflammation and are described elsewhere (Salter and Stevens, 2017). Microglial cells express numerous pattern-recognition receptors (PRRs) such as TLR1/2, TLR3, and TLR4 (reviewed in Colonna and Butovsky, 2017). Many other immune receptors are also expressed in microglial cells including CD4+, chemokine receptors, receptors which regulate the activation of microglial cells such as Triggering Receptor Expressed on Myeloid cell 2 (TREM2). These receptors might serve as a marker of inflammation during the course of HIV-1 infection (Gisslén et al., 2019). Many receptors for pro-inflammatory factors (TNFα, IL1β, IL10) and anti-inflammatory factors (IFNα/β) are also expressed (Lee et al., 2002). Interestingly, released anti-inflammatory factors transfer signals to the cell interior via a specific receptor complex to generate an antiviral response (Taniguchi and Takaoka, 2002). An exhaustive review of the wide range of immune receptors is described elsewhere (Colonna and Butovsky, 2017). Apart its function in immune surveillance to eliminate the microbes, microglial cells can also eliminate dead cells, redundant synapses, protein aggregates such as amyloid plaques (reviewed in Colonna and Butovsky, 2017; Salter and Stevens, 2017; Sominsky et al., 2018). These cells are involved in several functions of the CNS, including neurogenesis, the control of synaptic density and connectivity and the regulation of synaptic plasticity through the release of many cytokines such as Brain-Derived Neurotrophic Factor (BNDF). Of note HIV-1 Tat protein can inhibit BNDF by up regulating the microRNA mir-34a (Santerre et al., 2019). Microglial cells can also engulf neurotransmitters released in excess and therefore prevent excitotoxicity and participate in the cross talk between microglial cells and astrocytes. Due to their multifunction, any dysfunction in microglia cells will lead to many diverse diseases including brain aging or neurodegenerative diseases such as Alzheimer disease, Parkinson disease and amyotrophic lateral sclerosis. They are also central players in the occurrence of pathological neuroinflammation observed in patients under cART. HAND is thought to be related to excessive secretion of cytokines and chemokines (Yadav and Collman, 2009; Burdo et al., 2013; Eggers et al., 2017). In a recent study the gene expression profile in microglial cells from infected patients under cART with or without HIV encephalitis (HIVE) has been investigated which showed differential expression of several classes of genes compared to non-infected patients. 64% of the genes were effected in HIVE positive patients and 24% in HIVE negative patients (Ginsberg et al., 2018). This report strongly suggests that neuroinflammation, which leads to neurodegenerative diseases such as HAND, is associated with microglial impairment. Neuroimaging such as positron emission tomography (PET) has shown that neuroinflammation develops even under effective cART (Tavazzi et al., 2014; Vera et al., 2016). This approach has highlighted the importance of microglial activation in HIV+ patients under efficient cART with or without any cognitive impairments. PET also allows to follow the level of microglial activation by targeting translocator protein (TSPO) (Sinharay and Hammoud, 2019). Indeed, upregulation of TSPO during microglial activation is related to the degree of neuroinflammation (Venneti et al., 2004). Activation of microglial cells will further lead to the progression of neurodegenerative diseases by impacting the BBB (reviewed in da Fonseca et al., 2014; Osipova et al., 2018). Indeed microglial cells activation modulates tight junctions of endothelial cells of the BBB (Sumi et al., 2010). Of note, inhibition of microglial activation by minocycline is associated with preservation of BBB integrity *in vitro* and with reduced disruption of the BBB *in vivo* (Yenari et al., 2006; Sheng et al., 2018).

In conclusion, microglial cells fulfill several criteria of a brain reservoir. Most importantly they can subsist for a very long time in the brain and they can colonize the brain parenchyma. Contrary to other potential reservoirs in the brain, these cells divide slowly expanding the viral reservoirs in the brain and thus allowing virus persistence and reseeding of the blood. They are also involved in many functions including immune surveillance. As a consequence, any dysfunction of these cells might explain the occurrence of HAND.

### Evidence Supporting That Microglial Cells Are Susceptible to HIV-1 Infection and They Contribute to the Formation of a Cell Reservoir in the Brain

It is believed that microglial cell infection arises from transmigration of infected monocytes occurring very early in the course of infection. Recently, a specific subset of infected monocytes which preferentially cross the BBB, the HIV+ CD14+ CD16+ monocytes, has been characterized (Veenstra et al., 2017). These cells express abundantly junctional proteins such as Junctional Adhesion Molecule-A (JAM-A) and Activated Leukocyte Cell Adhesion Molecule (ALCAM) and chemokine receptors CCR2 which help these cells to cross the BBB. In turn these infected monocytes may infect microglial cells. Alternatively, but still debated, infected CD4+ T cells migrating into the brain might be ingested by microglial cells (Murooka et al., 2012). Although it has not been clearly demonstrated, this later mechanism could be more efficient to spread the virus than exposure to the free virus (Baxter et al., 2014). Whatever the mechanism of infection, it appears that brain microglial cells are permissive to HIV-1 infection. This is despite high level of the restriction factor SAM domain and HD domain 1 (SAMHD1) (Rodrigues et al., 2017). The absence of restriction by SAMDH1 is due to its phosphorylation by the cyclin kinase 1 (CDK1) which is induced in microglial cells that cycle between G0 to G1 state (Cribier et al., 2013; Mlcochova et al., 2017). There is now evidence supporting that microglial cells are infected by HIV-1 both *in vitro* and *in vivo* (reviewed in Joseph et al., 2015). Previous studies from autopsy have identified HIV-1 DNA, RNA and protein in microglial cells (Cosenza et al., 2002; Churchill et al., 2006). However, it was pointed out that these patients died from severe form of HAND. A recent study confirmed that microglial cells are infected in patients whose viral level is suppressed but died from an HIV-1 unrelated outcome (Ko et al., 2019). In this study the authors used a unique cohort from the National Neuro AIDS Tissue Consortium (NTTC) which comprised 16 patients on cART with well-documented, sustained control of HIV-1. They used highly specific technology to detect and quantify both HIV-1 DNA and RNAs at the cellular level. Very interestingly they showed that both perivascular macrophages and microglial cells but not astrocytes harbored HIV-1 DNA. In 6 out 16 cases they also found HIV-1 RNA in these cells when HIV-1 RNA was undetectable in the cerebro Spinal Fluid (CSF) and in the blood. This result strongly argues in favor that virus production can take place in the CNS. Other studies have also shown that microglial cells are susceptible to infection *in vitro*. Several *in vitro* models of human microglial cells susceptible of infection have been developed (Janabi et al., 1995; Nagai et al., 2001; Garcia-Mesa et al., 2017; Rawat and Spector, 2017; Dello Russo et al., 2018). Some models for latency have been derived from these previous models and constitute valuable tools to study the mechanism of infection and the molecular mechanisms underlying the establishment and maintenance of HIV-1 latency in microglial cells (Garcia-Mesa et al., 2017; Alvarez-Carbonell et al., 2019). Evidence that the virus was detected in the CSF in patients under effective cART who had otherwise undetectable plasma HIV-1 also argues in favor for the production of HIV-1 in the brain (Edén et al., 2010, 2016; Ferretti et al., 2015). In addition, phylogenetic analyses also suggest an important compartmentalization of HIV-1 in the CSF even early in infection (Salemi and Rife, 2016; Bavaro et al., 2019).

Finally, infection of microglial cells is clearly demonstrated in animal models such as the macaque and the humanized mouse (reviewed in Kumar et al., 2016; Marsden and Zack, 2017; Gama et al., 2018; Abreu et al., 2019). In these models HIV-1 DNA, RNA and protein were detected in the brain. Importantly, studies on these models showed that the viral reservoir is established very early at 3 days post infection (Whitney et al., 2014). More interestingly, there is evidence that microglial cells can be latently infected (Avalos et al., 2017; Gama et al., 2017). A macaque model has recently been designed and a mechanism for the establishment of HIV-1 transcription suggested (Barber et al., 2006). In this model, microglial cells can be reactivated in response to cytokine stimulation (Qin et al., 2015; Xu et al., 2017). Interestingly, in a cell model developed in Karn's laboratory, the glucocorticoid receptor and the toll-like receptor 3 appeared to be crucial receptors for HIV-1 activation (Alvarez-Carbonell et al., 2017, 2019). In brain autopsies from patients whose infection was controlled HIV-1 DNAs in microglial cells and macrophages were also detected (Thompson et al., 2011; Desplats et al., 2013). Humanized mouse models were generated in which microglial cells were infected by HIV-1 *in vivo* (Asahchop et al., 2017; Llewellyn et al., 2018; Mathews et al., 2019; Su et al., 2019). These models will allow us to study the pathophysiology of microglial cell activation and to develop strategies aiming to reduce the pool of these reservoirs.

Altogether, there are now numerous evidence supporting that microglial cells constitute a major cellular reservoir in the brain. Perivascular macrophages might also be considered as a cellular reservoir but not a true lifelong existing one. Moreover, we might expect to reduce drastically this pool by intensifying cART or by preventing the transmigration of infected monocytes into the brain. Concerning astrocytes it is still debated whether they constitute a true reservoir (Al-Harti et al., 2018; Ko et al., 2019).

Thus, a complete understanding of the molecular mechanisms underlying establishment and persistence of HIV-1 latency in microglial cells is needed in order to design original strategies aiming to target these reservoirs.

## Establishment and Persistence of HIV-1 Latency in Microglial Cells

Infection and the constitution of a microglial cell reservoir might occur very early in the evolution of HIV-1 infection (Whitney et al., 2014). Some features of the microglial cells allow for the persistence of HIV-1 in the brain. When infected they are far more resistant to cytopathic effects and on the contrary of CD4+ T cells they are non-lytic. In addition, these cells are resistant to apoptosis. The mechanism of apoptosis resistance to HIV-1 deserves considerable attention since it helps to design alternative strategies based on the increase of apoptosis susceptibility (Kumar et al., 2014). Indeed, strategies aiming to reactivate HIV-1 from infected cells should consider the problem of resistance to apoptosis since the reinforced therapy might not be efficient against the newly productive but non-apoptotic cells. Finally, the antiretroviral drug efficiency is drastically reduced in anatomical and pharmacological sanctuaries such as the brain where microglial cells are mostly found and thus contributing also to viral persistence (Asahchop et al., 2017; Honeycutt et al., 2017).

**Figure 1:**
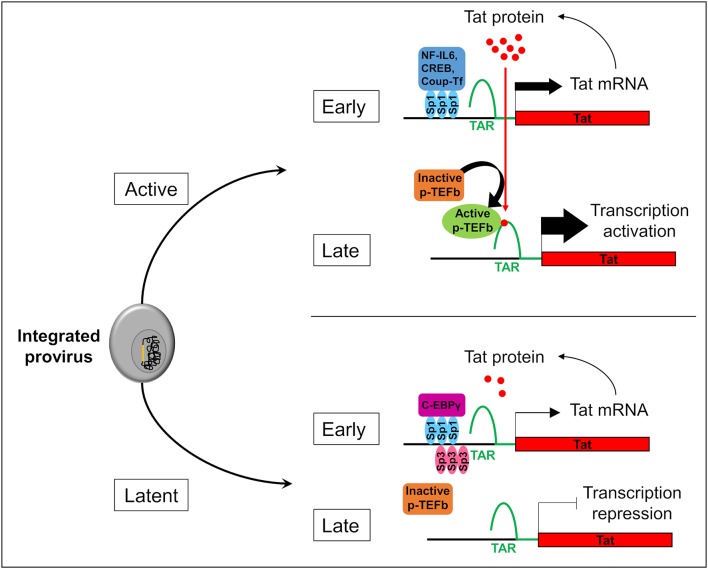
Stochasticity of transcription in microglial cells. The provirus (in yellow) integration in the genome of the microglial host cell (in gray) leads to two possible fates of the cell: active or latent. In active cells, Sp1 allows the anchorage of transcriptional activators like NF-IL6, CREB, and Coup-Tf. It enhances Nf-κB activity and allows the transcription of the viral transactivator Tat in the early phase of transcription. Then, Tat promotes the recruitment of active p-TEFb elongation complex at TAR regions. This recruitment allows viral RNA elongation and transcription activation during the late phase. It results a Tat production which promotes a Tat positive feedback loop and enables a strong transcription of the HIV-1 promoter. In the early phase in latently infected microglial cells, Sp3 act as a transcriptional repressor. In addition, C-EBPγ competes with the transcriptional activator NF-IL6. These repressors reduce Tat expression, p-TEFb recruitment, and viral RNA elongation during the late phase of transcription. If Tat production is under a threshold level, transcription is inhibited and the virus is inactive.

Two forms of latency have been described which explain HIV-1 persistence in microglial cells. Latency which occurs before the integration of the HIV-1 genome into the host is called pre-integration latency. This form of latency has been frequently observed in CD4+ T cells and in cells of the monocyte-macrophage lineage (discussed in Le Douce et al., 2010). In the latter cells, pre-integration latency might constitute a viral defense mechanism which could lead to the establishment and persistence of HIV-1 infection (Cara and Klotman, 2006; Hansen et al., 2016). This type of latency has not been described in microglia yet. We call post integration latency when HIV-1 genome has been incorporated into the host DNA genome. The integrated HIV-1 provirus can then be in an active-replication state enabling strong transcription of the HIV-1 promoter or in an inactive-state with a marked repression of HIV-1 transcription (Pai and Weinberger, 2017; Cao et al., 2018). In CD4+ T cells, latency is a very rare event with 1 to 10^6^-10^7^ cells being latently infected (Weinberger and Weinberger, 2013). This rare event has not yet been described but might occur in the other reservoirs infected with HIV-1. Mechanisms involved in CD4+ T cell latency are well-described (Khoury et al., 2018). The strength of the HIV Tat positive feedback loop regulates principally the viral fate selection (Figure 1) (Pai and Weinberger, 2017). In other word, there is stochasticity in HIV-1 transcription which explains that each single cell differs in many parameters (stage of cell cycle, level of expression of transcriptional activators/repressors, metabolic state…). Any mechanism reducing the level of HIV-1 Tat expression under a threshold level might therefore facilitate the establishment of latency (Karn, 2011). Integration of the viral genome into repressive heterochromatin environment (Jordan et al., 2001), transcriptional interference (Han et al., 2008), the lack of host transcriptional activators (Le Douce et al., 2010; Churchill et al., 2015), the presence of host transcriptional repressors (Le Douce et al., 2010; Churchill et al., 2015) or the influence of viral miRNA on the chromatin environment (Wang et al., 2009) are thought to favor the establishment of HIV-1 latency since all these mechanisms significantly decrease the strength of the Tat positive feedback. Of note, the mechanism involving viral miRNAs is however controversial and deserves further studies (Balasubramaniam et al., 2018). Additionally, post-transcriptional mechanisms regulating reactivation from latency have also been proposed (Sarracino et al., 2018). On the other hand, in microglial cells host transcription factors that facilitate the establishment of latency have been described. Intriguingly, it was observed that a new CNS strain of the virus has evolved with specific promoters (the 5' long terminal repeat or 5′ LTR) probably due to the compartmentalization of the virus in the brain (Churchill et al., 2015; Gray et al., 2016). Moreover, in contrast to other reservoirs, the NF-κB and the core region including Sp1 sites are sufficient for HIV-1 transcription in microglial cells (Rohr et al., 2003b). In particular, Sp1 proteins allow the anchorage of many transcriptional activators such as NF-IL6 also referred as liver-enriched transcriptional activator protein (LAP), CREB and Coup-Tf (Figure 1). Unlike CD4+ T cells which express only Sp1, microglial cells express both Sp1 and Sp3 which operate as transcriptional repressors. In addition, we and others have shown that in macrophages and microglial cells, a truncated form of NF-IL6, liver-enriched transcriptional inhibitory protein (LIP) and/or C-EBPγ is expressed and acts as repressor by competing with the transcriptional activator NF-IL6 (Schwartz et al., 2000) and (Tesmer and Bina, 1996). Noteworthy, this latter mechanism explains HIV-1 latency in microglial cells of a macaque model (Barber et al., 2006). Epigenetic regulation is also involved in the establishment of latency. Coup-TF interacting protein 2 (CTIP2 also known as BCL11b) is a key factor in promoting the formation of heterochromatin, the compact-inactive form of the promoter, in microglial cells. Indeed, CTIP2, apart its crucial role in establishing latency, is also involved in the persistence of latency by preventing HIV-1 reactivation, and in the generation of a cell microenvironment favorable to the establishment of HIV-1 latency. In addition, CTIP2 is described as an anti-apoptotic protein (Kamimura et al., 2007) and thus might also be involved in the apoptosis resistance in microglial cells (Le Douce et al., 2010; Kumar et al., 2014). Interestingly a new mechanism to prevent apoptosis in infected macrophage/microglia cells which survived the infection and became latently infected has been described (Castellano et al., 2017). They notably showed that apoptosis is blocked at a very early step and that the pro-apoptotic protein BIM which accumulate into mitochondria might serve as a new bio-marker of latently-infected macrophage/microglia cells *in vivo*.

CTIP2 is a Zinc finger transcription regulator which has been implicated in many functions required during development and in adult animal (reviewed in Le Douce et al., 2014). Germline disruption of CTIP2 is mortal stressing its importance during development. In fact, the essential role of CTIP2 in T cell development, odontogenesis, skin development and neuronal development are now well-documented (Le Douce et al., 2014). It was shown that CTIP2 is a transcriptional regulator binding directly on a promoter specific sequence (Avram et al., 2002) or binding indirectly to other promoter-bound transcription factors such as Sp1 (Avram et al., 2000).

**Figure 2:**
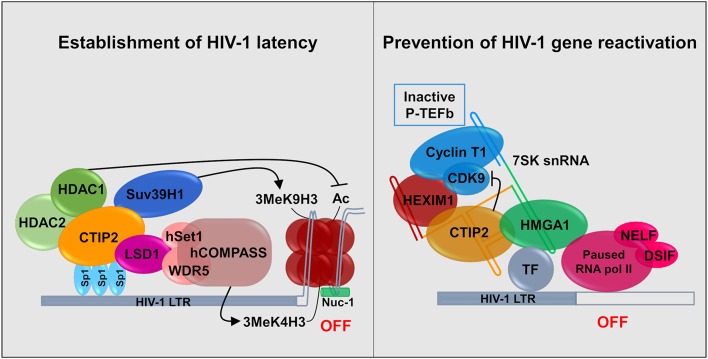
Establishment and persistence of HIV-1 latency in microglial cells: CTIP2 as a central actor. **(Left)** CTIP2 is involved in the establishment of HIV-1 latency by recruiting a chromatin modifying complex at the viral promoter (HIV-1 LTR). CTIP2 is anchored at Sp1 sites on the viral promoter and act as a scaffolding for the recruitment of several chromatin modifying proteins. They perform epigenetic modifications of histones of the nucleosome located at the promoter. CTIP2 recruits simultaneously HDAC2, HDAC1 and the HMT Suv39H1. HDAC1 deacetylate histones while Suv39H1 generates the trimethylation of H3K9 (H3K9me3). In addition, LSD1 promotes the trimethylation of H3K4 by the hCOMPASS methyltransferase complex through hSet1 and WDR5 recruitment. These two epigenetic marks are sufficient to promote chromatin compaction (heterochromatin) and inhibit transcription. **(Right)** CTIP2 prevents transcription restart by sequestrating the elongation factor pTEF-B in an inactive complex. 7SK snRNA complex is recruited at promoter via HMGA1 by interaction with transcription factors (TF). HMGA1 then recruits CTIP2 associated with the inactive form of PTEF-B (Cyclin T1 and CDK9) and HEXIM1, on the 7SK snRNA. Transcription is inhibited at two levels. First, CTIP2 inhibits the kinase activity of CDK9, a pTEF-B subunit which prevent RNA pol II activation by inhibiting the phosphorylation of RNA pol II and the two subunits NELF and DSIF. Second, the persistence of inactive pTEF-B at the promoter prevents any recruitment of active pTEF-B.

CTIP2 is involved in the establishment of HIV-1 latency by favoring the formation of heterochromatin in the vicinity of the viral promoter. It serves as a platform anchoring a chromatin modifying complex (Marban et al., 2007). Indeed, our work showed that in the presence of CTIP2 histone deacetylases HDAC1, HDAC2, and histone methyltransferase (HMT) SUV39H1are simultaneously recruited on the viral promoter which generates the H3K9me3 epigenetic mark (trimetylation of H3K9) (Figure 2). This work was the first to demonstrate the recruitment of HMT at the latent HIV-1 promoter. It paved the way for reactivation strategies that use a combination of molecules targeting multiple enzymatic activities. Furthermore, we characterized a new factor working in synergy with CTIP2 i.e., the lysine specific demethylase 1 (LSD1), which we showed to be a key factor in the molecular mechanisms of establishment of HIV-1 latency (Le Douce et al., 2012). In particular we established that LSD1 inhibits HIV-1 transcription and viral expression by recruiting hSet1 and WDR5 on the HIV-1 promoter, two members of the hCOMPASS complex, which induce H3K4me3 epigenetic mark (trimetylation of H3K4) (Figure 2). The association of H3K4me3 and H3K9me3 epigenetic marks might represent a new level of eukaryotic gene regulation. It was suggested that gene repression linked to H3K4me3 prevents the expression of cryptic promoters (Pinskaya and Morillon, 2009). This is supported by the findings that HIV-1 better integrates in active genes and thus can be considered as a cryptic gene (Wang et al., 2007).

Recently, we showed that CTIP2 is in another complex, which is able to counteract HIV-1 reactivation. In the initial report it was suggested that CTIP2 represses, the Tat-dependent late phase of HIV-1 transcription (Rohr et al., 2003a). Later experiments showed that an inactive form of the elongation factor pTEFb is recovered in a complex containing the non-coding 7SK snRNA and the proteins CTIP2 and HEXIM1 are attached to viral and cellular gene promoters in the absence of the HIV-1 factor Tat (Cherrier et al., 2013). pTEFb is a dimer comprising a regulatory subunit CyclinT1 and a catalytic subunit CDK9. CDK9 is a kinase which phosphorylates the Ser2 residue of the carboxyl terminal end of the RNA polymerase II and the negative transcriptional elongation factors NELF and DSIF. We showed that in this complex CTIP2 significantly inhibited CDK9 kinase activity thus inhibiting pTEFb function (Cherrier et al., 2013). In a following paper, we showed that the cellular protein High Mobility Group AT-hook 1 (HMGA1), which also belongs to the 7SK snRNA complex, recruited the inactive CTIP2/pTEFb complex onto the HIV-1 and cellular target promoters thus regulating their gene expressions (Eilebrecht et al., 2014). HMGA1 has been previously shown to interact with TAR to modulate Tat-dependent HIV transcription (Eilebrecht et al., 2013). It seems that HMGA1 is critical for the repressive function of CTIP2. We propose a model in which HMGA1 facilitates the recruitment of CTIP2-inactivated P-TEFb onto cellular and viral gene promoters (Figure 2).

As suggested above, CTIP2 has an important role in the regulation of the expression of many infected cells genes. Microarray analysis of a microglial cell line knocked down for CTIP2 showed up or down regulation of several genes (Cherrier et al., 2013). Among them the cellular cyclin-dependent kinase inhibitor CDKN1A/p21^waf^ was up regulated. When recruited to the p21 promoter, CTIP2 represses p21 gene transcription with the same mechanism described for the HIV-1 promoter (Cherrier et al., 2009). The p21 gene repression may favor HIV-1 latency since activation of p21 gene stimulates viral expression in macrophages (Vazquez et al., 2005). In addition, CTIP2 counteracts HIV-1 Vpr which is required for p21 expression. Interestingly we recently showed that HIV-1 Vpr mediates the depletion of the cellular repressor CTIP2 to counteract viral gene silencing (Forouzanfar et al., 2019). Altogether our findings strongly support the findings that CTIP2 generates a cellular environment which is favorable for HIV-1 latency. Furthermore, our results support that CTIP2 is a major actor involved in HIV-1 latency in microglial cells. In agreement with our findings, CTIP2 colocalized with the microglial marker Ionized calcium binding adaptor molecule 1 (Iba1) in latently infected patients but not in HIV encephalitis (HIVE) patients (Desplats et al., 2013). Moreover, the levels of CTIP2 and markers of heterochromatin such as HDACs and heterochromatin 1 (HP1) were increased in microglial cells from HIV-1 positive latent cases. This work and others suggested that CTIP2 can be used as an HIV brain latency biomarker (Cysique et al., 2019).

Recently we have identified and characterized a new cellular factor i.e., Hypermethylated in Cancer 1 (HIC1). This factor regulates together with CTIP2 and HMGA1 cellular and HIV-1 gene transcriptions (Le Douce et al., 2016). However, the repressive activity of the complex requires the deacetylase activity of Sirtuin 1 (SIRT1) in microglial cells. We showed that HIC1 interacted and cooperated with HMGA1 and modulated Tat dependent HIV-1 transcription. However, intriguingly it occurs in the presence of Tat but it is independent of the elongation factor PTEFb. The need for HMGA1 to interact with HIC1 to repress the Tat dependent HIV-1 transcription might suggest a new role for HMGA1 in this process (discussed in Le Douce et al., 2016). Our findings support the idea that the TAR element serves as a HIC-1 reservoir to promote HIC-1/Tat interaction.

We are far from elucidating all the molecular mechanisms which underlie HIV-1 latency in microglial cells. Further investigation in this field is needed in order to identify new potential targets in HIV-1 therapy.

## Targeting the Microglial Cells

Targeting all the HIV-1 reservoirs including the microglial cells in the brain is important in order to achieve either a sterilizing or a functional cure. Indeed, these cells are potential sources of HIV-1 reseeding in the blood. In addition, production of the virus in these cells has been associated with HIV-1 resistance and the development of HAND. However, targeting these cells which are located in anatomic and pharmacologic sanctuaries might be very challenging (Marban et al., 2016). Most importantly the access of drugs used in cART is limited by the blood brain barrier. Poor access of the drugs contributes to the persistence of HIV-1 in microglial cells (Cosenza et al., 2002). Another limitation is the existence of a residual neuroinflammation which is responsible for the occurrence of HAND in up to 50% of HIV-1 infected patients. A main concern is to prevent deleterious neuroinflammation associated with infected microglial cells (Rock and Peterson, 2006). To date, three strategies are used to target infected microglial cells (Veenhuis et al., 2019): (i) the Shock and Kill strategy, (ii) the Block and Lock strategy, and (iii) gene therapy. We will discuss in the following section the principles of these three approaches and outline their limitations. Finally, we briefly discuss how these limitations can be circumvented and how to rationalize treatments aiming the eradication or reduction of the pool of latently infected microglial cells.

### Strategies to Struggle Latently-Infected Microglial Cells

#### The Shock and Kill Strategy

**Figure 3:**
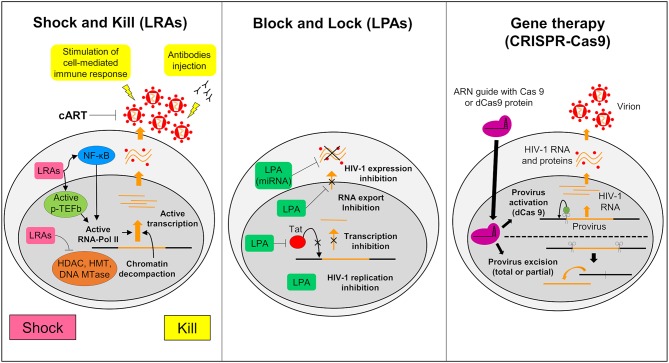
Strategies to struggle latently-infected microglial cells. The shock and kill: the strategy is based on the reactivation of virus expression, in order to eliminate reservoirs and to target latently-infected cells. Latency reversing agents (LRAs) are investigated to reactivate transcription for the “shock.” A first family of LRAs are HDAC, HMT, or DNA Mtases inhibitors and target epigenetic mechanisms. These LRAs induce chromatin decompaction and promote virus transcription. Another family of LRAs induce the expression of positive cellular factors (e.g., NF-κB and pTEFB) and/or their recruitment under their active form. These LRAs promote ARN pol II recruitment and favor transcription. The cART is maintained during this phase to clear reservoir without virus propagation in other cells. The “kill” can be enhanced by stimulation of the cell-mediated immune response or by using neutralizing antibodies and/or engineered antibodies. The block and lock: this strategy relates on the induction of a state of deep-latency which prevents any HIV-1 transcription. Several latency-promoting agents (LPAs) are investigated. They inhibit various step of virus expression like replication, transcription by Tat inhibition and RNA export. One promising LPA is a miRNA which inhibit virus expression. The gene therapy (CRISPR-Cas9): this strategy is based on the use of molecular scissors to excise total, or more reasonably, partial sequence of the provirus. Another strategy would be to use deficient Cas9 (dCas9) to carry reactivators or repressors of HIV-1 expression.

The Shock and Kill strategy is based on the reactivation of the latent virus. In this strategy the intensification of cART aims to clear the reservoirs either by the cytopathic effect of the reactivated virus or by the immune system via the actions of cytotoxic T cells (CTLs) (Schwartz et al., 2017). Latency reversing agents (LRAs) used in the reactivation target identified and characterized cellular factors involved in HIV-1 latency (Figure 3). Many LRAs are now used *ex vivo* and in clinical trials. However, the main targets are circulating CD4+ T cells and not the microglial cells (Kumar et al., 2015; Spivak and Planelles, 2018). There are currently 160 compounds used as LRAs which belong to two main families or to a third one which includes uncommon drugs with unique or unknown mechanisms (e.g., disulfiram and ixazomid) (Abner and Jordan, 2019). Screening of new LRAs is still a field of intensive research (Richard et al., 2018).

One family of LRAs targets epigenetic mechanisms that occur in latency (Darcis et al., 2016). This class comprises HDAC inhibitors (HDACi) such as Valproic acid, Vorinostat, panobinostat, and romidepsin; Histone methyl transferases inhibitors (HMTi) such as chaetocin and BIX 01294; and DNA methylation inhibitors such as 5-AzadC.

In the second family T cell activating agents are listed. These drugs induce the expression of positive cellular factors and/or their release from inactive complex (Jiang and Dandekar, 2015; Rice, 2016). Prostratin, bryostatin, and ingenol B by activating the PKC pathway release NF-KB and pTEFb from inactive complexes and increase pTEFb expression (Sung and Rice, 2006; Fujinaga et al., 2012; Pandeló José et al., 2014) which leads to HIV-1 reactivation (Darcis et al., 2015). Bromodomain inhibitors such as JQ1 and others are able to release pTEFb from the BRD4-pTEFb complex (Bartholomeeusen et al., 2012; Li et al., 2013; Darcis et al., 2015). Many other compounds including TLR agonists, TNFα, cytokines, and antibodies are also used as LRAs. A comprehensive review of these compounds are described elsewhere (Abner and Jordan, 2019).

However, these drugs used alone failed to fully reactivate HIV-1 expression *ex vivo* (Cary et al., 2016; Darcis et al., 2016). This might reflect the multi factorial mechanisms involved in promoting latency and the stochastic nature of latency (Sengupta and Siliciano, 2018). Another important inability of LRAs relies in their lack of specificity and to the heterogeneity of the reservoirs (Ait-Ammar et al., submitted). Indeed, the LRAs are mainly used in clinical assays to target circulating CD4+ cells but not the other reservoirs such as microglial cells. Therefore, combination of LRAs is now used to circumvent these problems. We expect synergistic effect which improves the efficiency of reactivation and reduces toxicity because of the lower doses used. Moreover, a recent report demonstrated the importance of the timing of the LRA combination treatment in the reactivation of HIV (Bouchat et al., 2016).

Reactivation of reservoirs should be followed by the elimination of the virus (Kim et al., 2018). There is now evidence that eradication of latently-infected reservoirs in patients involves humoral and cell mediated immune responses (Schwartz et al., 2017). Humoral immune response plays an important role in controlling HIV infection (reviewed in Ferrari et al., 2017). Over the past years, a new class of antibodies, called broadly neutralizing antibodies, have been shown to neutralize a wide range of HIV strains (Halper-Stromberg and Nussenzweig, 2016). Preclinical studies in which SHIVs infected macaque monkeys and HIV-1 infected humanized mice were treated by antibodies gave promising results (Halper-Stromberg and Nussenzweig, 2016). The efficacy of the treatment was even enhanced when various broadly neutralizing antibodies in combination was applied (Bruel et al., 2016) or when using multi-specific engineered antibodies like bi and tri-specific antibodies (Ferrari et al., 2016; Sun et al., 2016). Numerous strategies were proposed to enhance cell-mediated immune responses, e.g., CD8+ T cell or natural killer cell activities (Scully and Alter, 2016; Trautmann, 2016). Some methods aim to redirect HIV-specific cell mediated immune responses (Patel et al., 2016). In one strategy, redirected T cells recognizing a range of HIV antigens are expanded *ex vivo*. In another strategy genetically modified lentiviruses expressing artificial T cell receptors (TCRs) or chimeric antigen receptors (CARs) are used in the treatment (Patel et al., 2016). Increasing the specificity and the affinity of the epitopes of the receptors to achieve wider HIV epitope recognition is also realistic (Patel et al., 2016). The possibility to increase HIV-specific CD8+ T cell responses with heterocyclic peptides is currently tested. It is assumed that these peptides stimulate deeper the cell-mediated immune responses than native epitopes (Buhrman et al., 2013).

An original approach took advantage of the importance of co-stimulatory and co-inhibitory molecules involved in the regulation of T-cell responses (Sharpe and Abbas, 2006). Indeed, treatment by specific antibodies targeting the co-inhibitory molecule PD-1 is thought to reduce the size of the latently-infected reservoir and help to recover CD8+ T cell function from collapse. Finally, therapeutic vaccines might help CTLs to target HIV-1 infected cells derived from latent reservoirs (Deng et al., 2015).

#### The Block and Lock Strategy

An alternative approach to achieve long term control over HIV-1 infection without the need of cART is to induce a long lasting inhibition of HIV-1 gene expression (Figure 3) (Darcis et al., 2017). The molecules used to induce this process are called latency-promoting agents (LPAs). LPAs are expected to suppress the HIV-1 gene expression by inducing a deep latency state (the block) and thus preventing HIV-1 gene transcription (the lock) (Elsheikh et al., 2019). The first LPA identified from a marine sponge is a chemical derivative of corticostatin i.e., Didehydro-corticostatin (dCA). It inhibits Tat activity by preventing Tat-TAR interaction (Mousseau et al., 2015). To date many other LPAs have also been described (reviewed in Castro-Gonzalez et al., 2018). One of them, the ABX464, an inhibitor of Rev which is involved in the RNA export blocks HIV-1 replication *in vitro* as well as in animal models (Campos et al., 2015). Cellular mi RNAs are also potential molecules to be used in the Block and Lock strategy. It has been known for a long time that miRNAs are important modulator of HIV-1 latency (Huang et al., 2007). Screening and computational analysis have allowed the characterization of many miRNAs which impact HIV-1 transcription. One such promising miRNA, i.e., miRNA29a, which inhibits the virus transcription in Jurkat cells should be investigated for its effects in microglial cells (Hariharan et al., 2005). It is suggested that inducing miRNA29a expression in these cells could lead to suppression of HIV-1 gene expression.

#### The Gene Therapy

Gene therapy has been widely considered as a tool to combat HIV-1. It relies on nuclease-mediated gene editing tools such as the zinc fingers nuclease (ZFN1), the transcription activator-like effector nucleases (TALEN) and the CRISPR/Cas9 technologies (Panfil et al., 2018; Wang et al., 2018). Various gene therapies have been launched since the sterilizing cure of the Berlin patient (Allers et al., 2011). This patient received transplantation of hematopoietic stem cells from a donor who was homozygous for the CCR5-delta32 mutation (ΔCCR5) conferring resistance to HIV infection. Recently a second patient who shared similar medical contexts, the London patient, was also cured from HIV-1 infection (Gupta R. K. et al., 2019). From this example, the idea to build HIV-1 resistant cells has arisen (Barmania and Pepper, 2013). Clinical trials using ZFN targeting CCR5 are ongoing and deserve attention (Mehta et al., 2017). However, The CRISPR/Cas9 gene editing has become the tool of choice, since ZFN and TALEN are costly and time consuming. This tool uses a guided RNA and a Cas9 nuclease that excise DNA sequences of target specific cellular factors such as CCR5 or HIV-1 DNA thus eliminating the HIV-1 provirus (Figure 3). The first targeted DNA sequence by the CRISPR/Cas9 technology was the NF-KB binding site located in the HIV-1 LTR (Ebina et al., 2013). Since then, many other HIV-1 sequences have been targeted (Xiao et al., 2019). This technology may serve to redesign the gene expression of cells such as CTLs so that they target more specifically HIV-1 infected cells. It is believed that by this way the antiviral immunity system could be more efficiently boosted against HIV-1 infected cells and activated reservoirs producing new virions (Mehta et al., 2017). We might even use a variation of the CRISPR/Cas9 technology to reactivate latently infected cells or to induce deep latency (Wang et al., 2018). For this purpose, a defective Cas9 (dCas9) protein is fused to activators to reactivate latently-infected cells (Figure 3) or to repressors to suppress HIV-1 expression (Wang et al., 2018). *In vitro* trials suggested a potential application of CRISPR/dCas9 in the reactivation of latent HIV (Zhang et al., 2015). These trials showed reactivation of HIV expression in CD4+ T cells and in microglial cell lines (Cary and Peterlin, 2016). Interestingly, CRISPR/dCas9 when associated with HDAC inhibitors and PKC activators reactivated HIV in a synergistic manner (Limsirichai et al., 2016).

### Limitations and Challenges When Targeting Brain Microglial Cells

The current therapeutic strategies have limited efficacy to achieve cure. Their limitations comes from (i) the intrinsic nature of the strategy, (ii) the nature of the cells targeted, or (iii) the attributes of the virus to escape antiviral responses. Regarding microglial cells their specific cell properties and their location in anatomical and pharmacological sanctuaries make them difficult to target. In consequence, the presence of the BBB drastically reduces drug access to currently used drugs in cART (Asahchop et al., 2017). New antiviral drugs with improved CNS penetrance have been introduced in the last years (Veenhuis et al., 2019). However, some showed important side effects. For example, a new integrase inhibitor which showed important CNS uptake (Letendre et al., 2014) was neurotoxic showing severe neuropsychiatric effects (Scheper et al., 2018). Various technics improving delivery of drugs in the CNS and in the CNS-cell types are currently explored. Mechanisms underlying weak drug penetrance are mainly related to differential expression of efflux transporter and to multidrug resistance proteins (Valcour et al., 2011; da Fonseca et al., 2014). Several invasive and non-invasive methods for drug delivery are currently explored including modulation of brain barrier, ultrasound based BBB opening, endogenous transporter, nanoparticles, liposomes, dendrimers… (reviewed in Barnabas, 2019). Original route of drug delivery to the CNS are also explored such as drug delivery from the nose to the brain (Gupta S. et al., 2019).

We should also consider immune-based therapeutics to clear reservoirs activated by the LRAs. Animal models have shown the importance of the CTLs and neutralizing antibodies raised against HIV-1 in immune activation and clearing infected reservoirs in the CNS. Therefore it deserves considerable attention to ensure the feasibility of the Shock and Kill strategy (Brockman et al., 2015; Trautmann, 2016; Subra and Trautmann, 2019).

An important limitation of the use of the Shock and Kill strategy is that reactivation of microglial cells during the Shock therapy leads to neuroinflammation, since the release of pro-inflammatory factors and some viral proteins such as Tat and gp120 during the shock therapy are neurotoxic. Therefore, LRAs induced reactivation of reservoirs has to be associated with drugs preventing the adverse effects of neuroinflammation. One way to prevent inflammation is to improve cART via targeting HIV-1 transcription and/or by inhibiting RNA export in order to counteract the effects of the pro-inflammatory cytokines and to prevent synthesis of viral proteins (Schwartz et al., 2017). Molecules which have anti-inflammatory effects such as curcurmin are also investigated in microglial inflammatory responses (Chen et al., 2018). A new drug formulation using lipid nano-carrier and administration by intra nasal route are currently investigated (Vaz et al., 2017). Remarkably, many drugs targeting the NF-κB pathway (G1V001), the protein Tat (dCA, triptolides…) and rev (ABX4641) induce a deep latency and are thought to be used in the Block and Lock strategy as an alternative to the Shock and Kill strategy. We might use both strategies in a sequential time schedule. First, the Shock and Kill strategy which reactivates and decreases the pool of reservoirs. Followed by application of drugs exploiting the Block and Lock strategy, which suppresses transcription in persisting reservoirs and induces deep latency. Although, it is still an emerging field of research the Block and Lock strategy appears to be more than just an alternative to other strategies. Interestingly, less adverse effects with drugs targeting viral proteins are awaited. We believe that combination of the Block and Lock strategy with the Shock and Kill might allow the reduction of all HIV-1 reservoirs, including the microglial cell reservoir to a level, which does not need any further treatment to achieve a functional cure.

Gene therapy compared to a pharmacological approach such as the Shock and Kill strategy is still in its infancy but appears to be very promising. A recent review compares the advantages and weaknesses of these two approaches i.e., the pharmacological vs. CRISPR/Cas9-based shock strategies (Darcis et al., 2018). Gene therapy, in the same way as pharmacological approaches, does not resolve the problem of drug penetrance in the CNS where microglial cells are located. Indeed, currently used adeno-viruses in *in vitro* essays induce severe inflammation (Chew et al., 2016). Moreover, another important limitation of gene editing was discovered; HIV-1 can escape CRISPR/cas9 mediated suppression. Indeed, following an initial attempt to establish proof of concept of the use of gene editing targeting specifically HIV-1 (Kaminski et al., 2016), it soon appeared that the virus was able to subvert the DNA repair machinery to evolve quickly into CRISPR/Cas9 resistant strains (Liang et al., 2016; Wang G. et al., 2016; Wang Z. et al., 2016; Yoder and Bundschuh, 2016). On the other hand combination of guided RNAs targeting different HIV-1 specific sequences may drastically reduce the occurrence of HIV-1 escape (Wang et al., 2018). A major off-target of this gene editing tool is the increase of undesirable gene mutations and chromosomal translocations (Yee, 2016). Efforts in reducing these off-target effects are required in order to improve this elegant and attractive approach. For example, bioinformatics could help to select the correct guided RNAs that avoid unexpected DNA cleavage (Zhu et al., 2017). Another limitation of the use of gene editing technology is that it has been mainly tested *in vitro* and mainly in CD4+ T cells. Experiments conducted *ex vivo* showed that redirected immune cells reached only a tiny fraction of the HIV-1 reservoirs. Therefore, this technology does not make possible to drastically reduce the number of infected cells, which is required to achieve functional cure (Wang et al., 2018).

Rationalization of these strategies is required to optimize either of these strategies to target microglial cells. Since we cannot make brain biopsies due to ethical concerns, it is critical to design these strategies and test their feasibility in adequate animal models. Indeed, the efficacy and the pharmacokinetic and pharmacodynamic characteristics of drugs are tested only in preclinical studies. Non-human primates (NHP) and humanized mice are currently the best-suited animal models to study either strategies. However, there are many ways to evaluate the efficiency of a therapy and to evaluate HIV infection in the brain. The search of specific biomarkers for latently infected microglial cells may lead to the development of original strategies, which specifically target brain reservoirs. One such specific marker of the CD4+ T cell reservoir, CD32a, has been described (Descours et al., 2017), although its specificity is debated (Badia et al., 2018). Much research has been focused on finding new specific biomarkers for brain reservoirs, nonetheless their use will be very challenging. Indeed, it will be difficult to target brain reservoirs *ex vivo*, even though it is technically possible for blood cells, and very difficult to target them *in vivo* due to their paucity and localization in the entire brain parenchyma. For example, the invasive techniques used to evaluate the evolution of biomarkers in the CSF such as neopterin, CTIP2, TREM2, and NFL after a treatment cannot be performed too frequently (Jessen Krut et al., 2014). Non-invasive methods such as neuroimaging are therefore needed for brain evaluation (discussed in Garrido and Margolis, 2015). Comprehensive neuropsychological testing is also required to detect subclinical deficits (Haddow et al., 2013). It will be essential to evaluate the efficiency of each strategy that target microglial cells. For this purpose, highly sensitive methods have been developed such as the single copy assay (SCA) which allows the detection of HIV RNA in the CSF from infected patients on cART or from elite controllers whose HIV RNA level was initially undetectable in the plasma and the CSF (Dahl et al., 2013).

## Conclusion

HIV cure is theoretically possible but not yet feasible with current approaches. Complete cure demands targeting not only the CD4+ T cells reservoirs but also all the other potential reservoirs located in sanctuaries with low drug penetrance. This is particularly true for the microglial cells in the CNS which is considered as the main reservoir for HIV. Indeed, due to their properties (slow turn over, long half-life…), these cells are thought to persist lifelong. Other true reservoirs such as macrophages may be eliminated by intensifying cART and/or by targeting HIV infected monocytes which is the source of infection. Other reservoirs such as astrocytes or the newly identified pericytes (Bertrand et al., 2019) may constitute true reservoirs, but these are still debated and deserve far more investigations. Currently, three strategies are considered, which allow functional cure: two pharmacological approaches including the shock and kill strategy and the alternative block and lock strategy and the CRISPR/cas9 based technology, which is a gene editing tool based strategy. However, the Shock and Kill strategy is not suitable to eliminate reservoirs like microglial cells, since the reactivation of microglial cell reservoirs leads to neuroinflammation, which is the origin of HAND. The search for potential epigenetic regulators that control microglial cells are therefore needed. The most investigated epigenetic regulators are the long non-coding RNAs (Qu et al., 2019) and the mi RNAs (reviewed in Cheray and Joseph, 2018). However, due to the stochasticity of HIV-1 transcription the Shock and Kill strategy does not allow reactivation of all latently infected microglial cells. Alternative strategies are therefore considered. The Block and Lock strategy has the advantage that the risk of inflammation in the brain is drastically reduced. More importantly, this strategy since it targets transcription and/or RNA export might be used following the Shock and Kill strategy. The combination of strategies should lead to the decrease of the pool of the microglial reservoir. It may also induce deep latency in reservoirs that are not reactivated by LRAs. The gene editing tool, such as the CRISPR/Cas9 technology, because it operates on specific sequences is also very promising. Still in its infancy, this elegant approach can target either cellular factors, which are involved in HIV-1 resistance, or viral factors. This technology can be used either to reactivate the virus or to excise and eliminate the provirus from its host genome. However, several challenges have to be overcome such as *in vivo* delivery in the brain and long-term toxicity before its use in the clinic and thus needs far more investigations. An original way to deliver CRISPR/cas9 guided RNA across the BBB has been developed recently and hold promises (Kaushik et al., 2019). Notably, it was shown that this non-invasive method which uses magnetically guided delivery of RNAs inhibits latent infection of microglial cells and cross the BBB in *in vitro* models.

In conclusion, targeting microglial cells is crucial but might be very challenging. We have to keep in mind that these cells are resistant to the cytopathic effects of the virus and resistant to apoptosis. Thus strategies aiming to induce apoptosis in infected microglial cells have to be developed (Le Douce et al., 2010). It appears that the cellular factor CTIP2 (Bcl11b) has a pivotal role in the establishment and persistence of HIV latency, in the genesis of a favorable environment for HIV latency and in the resistance to apoptosis (Le Douce et al., 2014). CTIP2 silencing by RNA interference has been proposed in cancer since downregulation of CTIP2 leads to apoptosis in malignant cells but not in normal mature cells (Grabarczyk et al., 2007; Huang et al., 2007). We might also design drugs targeting CTIP2 interacting proteins specifically involved in HIV-1 latency and apoptosis resistance. Moreover, establishing a protein interaction network focused on CTIP2 interactions in microglial cells will allow the identification and characterization of new actors involved in HIV-1 latency and apoptosis resistance.

## Author Contributions

CS and OR wrote the review. CW designed the figures and wrote the legends. CW, MD, JV, FD, SD, VG, PM, AM, and CV participated in the writing of the review.

### Conflict of Interest

The authors declare that the research was conducted in the absence of any commercial or financial relationships that could be construed as a potential conflict of interest.
